# Robotic-assisted cochlear implant surgery

**DOI:** 10.1016/j.bjorl.2024.101530

**Published:** 2024-11-04

**Authors:** Vagner Antonio Rodrigues Silva, Arthur Menino Castilho

**Affiliations:** Universidade de Campinas (Unicamp), Faculdade de Ciências Médicas (FCM), Departamento de Otorrinolaringologia, Cirurgia de Cabeça e Pescoço, Campinas, SP, Brazil

## Introduction

Artificial Intelligence (AI) refers to the ability of a machine or program to mimic human intelligence and perform tasks such as reasoning, problem-solving, and learning based on the data offered. AI permeates many aspects of our daily lives, including games, personal assistants (such as Siri), and transportation. AI has begun to be used in medicine to improve patient care, paving the way for healthcare delivery.^1^

Machine Learning (ML) is a subset of AI that uses the data and historical information provided to the program repeatedly and recognizes patterns to improve the performance of tasks according to that data. Considered as an application of AI, ML algorithms include supervised, unsupervised, and reinforcement learning. ML is practically used in biometric care, facial recognition in healthcare, banking, Amazon Alexa, and voice help in various computer applications.

Deep Learning (DL) is a type of ML inspired by the architecture of neurons in the human brain. An artificial neural network is made up of several layers, through which data is processed. In DL, multiple datasets are considered simultaneously, undergoing multiple analyses and reprocessing for successive evaluations. Each analysis occurs within a distinct layer based on the previous layer's output.^1^

AI in surgery involves autonomous movements. Robotics and decision assistance are the two main areas where ML has been used most frequently in surgery. ML decision support systems are becoming increasingly prevalent for diagnosing conditions, predicting outcomes, and identifying surgical candidates and potential postoperative issues.^2^

Doubts arise regarding legal responsibility if the surgery has complications. Government agencies like *the Food and Drug Administration* do not have specific legal frameworks for robots with autonomous actions. In the European Parliament's resolution of 2017, with recommendations to the Committee on Civil Law Rules on Robotics on Medical Robots, they stated that it is vital to respect the principle of supervised autonomy of robots, according to which the initial planning of treatment and the final decision on its execution will always remain with a human surgeon. It is unlikely that human surgeons will be entirely replaced by an AI-controlled surgical robot intended to support, not replace, a surgeon's ability to make and execute decisions.

The autonomy that a surgical robot can achieve may be divided into six stages.^3^

**Level 0** – Only the surgeon controls the robot's movement. The surgical tools accurately mimic the surgeon's movements at the control interface.

**Level 1** – Virtual accessories to improve the view of the surgical site or active limitations to direct the surgeon's movement are the forms of help offered. The three main enabling technologies for Level 1 autonomy are tissue interface detection, tool tracking, and eye tracking.

**Level 2** – Robots are competent to perform specific surgical activities according to the guidelines given by the doctor. During the task's duration, the robot's control passes from the human operator to the machine. With the drill pointing toward of the intended trajectory, the surgeon moves the arm into the proper position. The drill then drills the hole automatically, keeping the endosteal membrane intact.

**Level 3** – Robots are given perceptual skills to plan and perform specific tasks, understand the surgical environment, and alter the plan. An example of this technology includes CI surgery, which involves drilling in the temporal bone, planned and performed by a robotic arm until the opening of the cochlea.

**Level 4 –** The robot can interpret pre and intraoperative data, create an interventional plan consisting of a series of actions, execute this plan on its own, and adjust it as needed. In the discrete control paradigm, the system is supervised by a surgeon.

**Level 5 –** Robots can perform surgeries without a human's help. Not yet reached

## Robotic-assisted ci surgery

Robotic ear surgery, especially cochlear implantation (CI), has been increasing. Accesses to the cochlea through a single perforation tunnel through the mastoid are presented, called “percutaneous cochlear implant *surgery*” (minCIS) or “*direct cochlear access*” (DCA). This can be achieved using image-guided surgery systems and custom surgical targeting systems. However, CI surgery can utilize robotic assistance in several steps during surgery:

1 Mastoidectomy and tympanotomy via DCA;
2 Minimally invasive cochleostomy via a robot-assisted drilling instrument;
3 Alignment for correct insertion of the electrodes in the basal rotation of the cochlea.
4 Electrode Array Insertion

Also known as “robotic-assisted CI surgery” (RACIS) it is reliable for improving structure preservation. These include instruments for manual or motorized insertion of the electrode array (EA), microdrills for “atraumatic” cochleostomies, malleable EAs or models to enhance their insertion, or augmented reality systems capable of guiding the surgeon during maneuvers within the cochlea.^4^

There are insertion devices already approved by international agencies, as well as robots that pierce the temporal bone, posterior tympanotomy, and even cochleostomy and insertion of the electrode array. These surgeries take longer than the conventional one, between 4 and 6 h in duration, and at least one intraoperative tomography is needed to check the progress of the surgery.

Intelligent arm-supported and hand-guided microdrill robotic systems and a force sensor have been developed recently. They can detect changes in force transients and stop at the interface between bone and soft tissue, preserving the endosteal membrane. Augmented reality-assisted procedures allow the surgeon to visualize, in real-time, previously identified structures of interest or superimposed on microscopic or endoscopic 3D surgical images, making the necessary information easily available.

## Advantages and disadvantages of new tools for ci surgery

Minimally invasive access to the middle ear through a tunnel while maintaining mastoid integrity may seem like an advantage over standard mastoidectomy due to its reduced invasiveness, but this has yet to be proven, especially in terms of the time required to make this access safely.

No clinical benefit in preserving residual hearing or improved speech performance has been demonstrated to date. Robotic procedures have lasted longer than conventional surgery. However, it should be remembered that times for traditional surgery were much longer in the early 90 s and then decreased with the increase in the number of procedures, the experience of surgeons, and subsequent changes in surgical technique. Robotic access systems require one or two intraoperative CT scans to ensure facial nerve safety. This implies an addition of ionizing radiation.

Currently, favorable anatomical conditions (mainly width of the facial recess and inner ear malformations) are among the enrollment criteria for implantation through DCA procedure, excluding a part of the population from this type of surgery.^5^ Another and perhaps more important issue is the cost of robotic devices. There are no studies on the cost analysis of using robotics for otological surgery. This issue needs to be addressed in the future, as current robots represent the most expensive devices in an ENT operating room and the cost/benefit ratio is likely to be unfavorable.

## Conclusion

Robotic technology will transform the surgical field. Robots now have autonomous and semi-autonomous modes due to the development of new features made possible by AI, ML, and DL. High-level autonomous capabilities are replacing the low-level automation of early medical robots in terms of task complexity. With few official restrictions and very controversial ethical issues, the legal and ethical ramifications of autonomous activities by robots remain a topic of discussion.

## Funding

The authors have no financial relationships relevant to this article to disclose.

## Declaration of competing interest

The authors declare no conflicts of interest.
